# CASMI—The Small Molecule Identification Process from a Birmingham Perspective

**DOI:** 10.3390/metabo3020397

**Published:** 2013-05-21

**Authors:** J. William Allwood, Ralf J.M. Weber, Jiarui Zhou, Shan He, Mark R. Viant, Warwick B. Dunn

**Affiliations:** 1 School of Biosciences, University of Birmingham, Edgbaston, Birmingham, B15 2TT, UK; E-Mails: j.w.allwood@bham.ac.uk (J.W.A.); r.j.weber@bham.ac.uk (R.J.M.W.); m.viant@bham.ac.uk (M.R.V.); 2 College of Biomedical Engineering and Instrument Science, Zhejiang University, Hangzhou 310027, China; E-Mail: jrzhou@me.com; 3 School of Computer Sciences, University of Birmingham, Edgbaston, Birmingham, B15 2TT, UK; E-Mail: s.he@cs.bham.ac.uk

**Keywords:** CASMI, metabolite annotation, metabolite identification, KEGG, ChemSpider, PUTMEDID-LCMS, MetFrag

## Abstract

The Critical Assessment of Small Molecule Identification (CASMI) contest was developed to provide a systematic comparative evaluation of strategies applied for the annotation and identification of small molecules. The authors participated in eleven challenges in both category 1 (to deduce a molecular formula) and category 2 (to deduce a molecular structure) related to high resolution LC-MS data. For category 1 challenges, the PUTMEDID_LCMS workflows provided the correct molecular formula in nine challenges; the two incorrect submissions were related to a larger mass error in experimental data than expected or the absence of the correct molecular formula in a reference file applied in the PUTMEDID_LCMS workflows. For category 2 challenges, MetFrag was applied to construct *in silico* fragmentation data and compare with experimentally-derived MS/MS data. The submissions for three challenges were correct, and for eight challenges, the submissions were not correct; some submissions showed similarity to the correct structures, while others showed no similarity. The low number of correct submissions for category 2 was a result of applying the assumption that all chemicals were derived from biological samples and highlights the importance of knowing the origin of biological or chemical samples studied and the metabolites expected to be present to define the correct chemical space to search in annotation processes.

## 1. Introduction

Metabolites are the building blocks for a range of chemicals (for example, proteins and DNA) and cellular components (for example, cell walls) in biological systems and are involved in many biological processes, including metabolism and regulatory processes (for example, allosterism and riboswitches). The untargeted and holistic study of the metabolite composition of biological samples (for example, cells and tissues) is defined as metabolomics and is applied to provide a sensitive and dynamic measure of the phenotype [1,2].These phenotypic data provide insights in to biological mechanisms [3,4] and can also act to define biomarkers related to, among others, environmental stress [5,6], disease [7] and drug toxicology [8].

The workflow in untargeted metabolomic studies starts with experimental design, progresses through sample collection, sample preparation and data acquisition to the final processes of data analysis and biological interpretation [9]. In these studies, samples are prepared and analysed typically without the chemical identity of metabolites in the sample being known *a priori*. In studies applying chromatography-mass spectrometry as the analytical platform, hundreds to thousands of metabolite features are detected where a single metabolite is related to multiple metabolite features (*i.e.*, multiple chemical derivatisation products in GC-MS [10] and different ion types in LC-MS [11]). Following univariate and multivariate data analysis, annotation or identification of metabolite features identified as biologically important is performed. This process is currently a large bottleneck in holistic metabolomics studies, and the capabilities have been reviewed (for example, see [12]). When applying chromatography-mass spectrometry studies, the first step of annotating metabolites is to apply the accurately measured mass-to-charge ratio (*m/z*) and match the feature to molecular formula(e) with the same *m/z* ratio (or associated mass of the non-charged metabolite) within a specified mass error (for example, see [13,14,15,16]). Where multiple molecular formulae are reported, further chemical rules can be applied (for example, the seven golden rules [17], which includes relative isotopic abundance calculations) to reduce the number of possible molecular formulae. These molecular formulae can be searched for in chemical (for example, ChemSpider [18] or PubChem [19]) or metabolite-specific (for example, KEGG [20], HMDB [21] or MetaCyc [22]) to report specific metabolites. The second step of annotation is to apply gas phase fragmentation (*i.e.*, MS/MS [23] or MS*^n^* [24]) of the molecular or related ion and to match experimental data to mass spectral libraries (for example, METLIN [25] or MassBank [26]) or to theoretical fragmentation patterns derived from open source software (for example, MetFrag [15]) or commercial software (e.g. Mass Frontier from HighChem [27]).

Four levels of reporting metabolite annotation and identification are available as defined by the Metabolomics Standards Initiative in 2007 [28]. These levels include identification (level 1), where two orthogonal properties of the metabolite are matched to the same properties of an authentic chemical standard analysed, applying the same analytical method. Levels 2 and 3 provide annotation as metabolites (level 2) or metabolite classes (level 3) by matching to data present in chemical or metabolite-specific databases and mass spectral libraries, but without comparison to authentic chemical standards analysed, applying the same analytical methods. Level 4 defines the metabolite as unidentified.

Here, we report our submissions to the CASMI open contest, specifically, eleven challenges in category 1 and in category 2 related to high resolution LC-MS data. We provide our final submissions, the workflow applied to arrive at our submissions and specific comments in relation to the contest.

## 2. Results and Discussion

### 2.1. Description of Methods Applied

A research team from The University of Birmingham competed in the CASMI open challenge, specifically categories 1 and 2 related to liquid chromatography-mass spectrometry. All challenges were performed with the exception of challenges 11, 12 and 16; these challenges were assessed, though, because of complexity in the data, specifically, in-source fragmentation, so it was decided not to submit responses. No results were submitted for categories 3 and 4 related to gas chromatography-mass spectrometry.

Workflows previously developed by one of the authors (W.D.) and colleagues were applied to compete in category 1. Workflows 1 and 2 of the PUTMEDID-LCMS workflow series [13] were applied to annotate different metabolite features as the [M+H]^+^ or [M−H]^−^ ions or as isotopic peaks (for example ^13^C and ^34^S), applying retention time (RT), correlation coefficient analysis, *m/z* differences and median peak areas. The molecular mass of the uncharged metabolite was calculated from these data and matched to a large reference file containing accurate molecular masses and their associated molecular formula (13,061 in total, derived from PubChem and containing the elements C, H, N, O, P, S, Br, Cl, F and Si). A mass tolerance range of 5 ppm was applied, unless stated otherwise. Where more than one molecular formula was reported, the relative isotopic abundances (RIA) for carbon and sulfur were calculated using response data and accurate mass differences to filter the number of molecular formulae.

The authors have an interest in performing annotation of metabolites not present in mass spectral libraries. We applied MetFrag [15] to construct *in silico* fragmentation patterns and compare these data to experimental MS/MS data, because MetFrag software is freely available. Here, the molecular formula or formulae reported in category 1 of the same challenge were inputted on a single and manual basis in to the on-line MetFrag software, followed by searching for the molecular formula in the KEGG and/or ChemSpider databases and reporting of all molecular structures with the defined molecular formula. In the second stage, *in silico* fragmentation of each putative molecular structure was performed applying MetFrag and matched to the experimental MS/MS data provided. The match scores provided by MetFrag were applied to report putative molecular structures after manual assessment by the authors to ensure that the match scores reflected the different structures reported.

### 2.2. Results

The processes followed to construct the results submitted to the CASMI open contest, for each challenge in categories 1 and 2, are described below. We describe the data provided for challenge 1 to inform the readers of the typical data available.

#### 2.2.1. Challenge 1

For challenge 1, four data files were available; (i) the MS1 raw data (mzXML and netCDF formats), (ii) the MS1 peak list (txt format), (iii) MS2 raw data acquired at three different collision energies (mzXML format) and (iv) the MS2 peak lists for each collision energy at which MS/MS data were acquired/applied. Information on the instrument applied to acquire the data, the mass resolution and expected mass accuracy and retention time were also provided to assist the contestants. Similar data and further information were available for all other challenges. The three *m/z* values defined in the challenge as being detected in positive ion mode were analysed, applying workflows 1 and 2 of the PUTMEDID_LCMS collection of workflows with a mass accuracy of 5 ppm (as defined in the experimental summary). The results showed that the [M+H]^+^ ion was detected and reported a single molecular formula of C_18_H_36_N_4_O_11_, which was present in the trimMMD_sortAmass.txt file applied in workflow 2. The correct molecular formula was submitted. This molecular formula and the fragmentation mass spectrum acquired at 30eV were submitted to MetFrag applying KEGG as the chosen database. MS/MS data were provided at three different collision energies, and the data acquired at 30eV was chosen, as these MS/MS data provided the greatest number of product ions to allow structural information to be deduced most accurately. Two metabolites were reported, Kanamycin A and C, with *in silico* fragmentation data matching to 10 experimentally derived product ions for the former and eight for the latter metabolite. As a greater number of product ions were matched for Kanamycin A, this metabolite was submitted as the molecular structure. The correct molecular structure was submitted.

#### 2.2.2. Challenge 2

The four *m/z* values defined in the challenge as being detected in negative ion mode were analysed applying workflows 1 and 2 of the PUTMEDID_LCMS collection of workflows with a mass accuracy of 5 ppm (as reported for the same instrument in challenge 1). The results showed that the [M-H]^−^ ion was detected and reported no matches to a molecular formula present in the trimMMD_sortAmass.txt file applied in workflow 2. The search was repeated with a mass accuracy of 10 ppm, but no matches were reported. The *m/z* of the uncharged metabolite (592.1969 Da) was manually calculated and submitted to MetFrag with a mass accuracy of 5 ppm, where applying the KEGG database provided no hits and where applying the ChemSpider database provided 193 hits related to twenty-nine possible molecular formula. When all molecular formula containing F, Cl, Si or Br were removed (as it was not expected that the correct metabolite would contain these elements), 12 molecular formula remained. The data showed no evidence for the presence of sulfur in the molecular formula (as defined by relative isotopic abundance), and eight molecular formula containing sulfur were removed to leave four molecular formulae. Applying the relative isotopic abundance for carbon showed that 29 carbons were present in the molecular formula and one molecular formula was removed (C_21_H_32_N_6_O_14_). Three molecular formula remained; C_32_H_32_O_11_, C_33_H_28_N_4_O_7_ and C_38_H_28_N_2_O_5_. The correct molecular formula was not submitted, as the experimentally derived mass error (>30 ppm) was greater than the mass error reported with the data and expected for the mass spectrometer applied. The CASMI organisers have now provided data following recalibration; this provides an accurate result as defined by them. Submitting the fragmentation mass spectrum acquired at 20eV (MS/MS data at one collision energy of 20eV were provided) to MetFrag and applying ChemSpider as the chosen database reported six metabolites, and these were submitted to the contest with the MetFrag reported scores. The correct molecular structure was not submitted, because the correct molecular formula was not applied. One important point was observed in this challenge; although the mass accuracy of a specific mass spectrometer can be reported as a specific ppm range (+/− × ppm), this may not always be true for a subset of metabolites (for example, with a low response or where ion statistics do not allow an accurate determination of peak shape and apex). 

#### 2.2.3. Challenge 3

The five *m/z* values defined in the challenge as being detected in negative ion mode were analysed applying workflows 1 and 2 of the PUTMEDID_LCMS collection of workflows with a mass accuracy of 5 ppm (as reported for the same instrument in challenge 1). The results showed that the [M-H]^−^ ion was detected and reported no matches to a molecular formula present in the trimMMD_sortAmass.txt file applied in workflow 2. The process was repeated with a mass accuracy of 10 ppm, and one molecular formula was reported, C_13_H_19_N_7_O_7_S_2_. On assessing the sulfur relative isotopic abundance, it was calculated that three sulfur atoms were present in the molecular formula and, therefore, that this molecular formula may be incorrect. The mass (or molecular weight) of the uncharged metabolite (449.0826 Da) was manually calculated, assuming a [M−H]^−^ ion was detected (448.0754 + 1.0077 − 0.00055) and submitted to MetFrag with a mass accuracy of 5 ppm, where applying the KEGG database provide one molecular formula, C_14_H_27_N_1_O_9_S_3_. This molecular formula matched to the experimental relative isotopic abundance for sulfur and was submitted. The correct molecular formula was submitted. Submitting the fragmentation mass spectrum acquired at 20eV (chosen from data acquired at four collision energies, as these MS/MS data provided the greatest number of product ions to allow structural information to be deduced most accurately) to MetFrag, applying KEGG as the chosen database and performing *in silico* fragmentation, reported a single metabolite, glucolesquerellin (6-methylthiohexyl glucosinolate), with three product ions being matched to *in silico*-derived fragmentation ions. This single metabolite was submitted to the contest. The InChI submitted to the contest did not match the correct InChI provided by the organisers. However, the InChI submitted to the contest almost matched the correct structure, differing only in the structural configuration of the hexose substructure.

#### 2.2.4. Challenge 4

The three *m/z* values defined in the challenge as being detected in positive ion mode were analysed applying workflows 1 and 2 of the PUTMEDID_LCMS collection of workflows with a mass accuracy of 5 ppm (as defined in the Experimental Summary). The results showed that the [M+H]^+^ ion was detected and reported a single molecular formula of C_16_H_21_NO_4_S. However, the relative isotopic abundance observed in the data showed no evidence of a sulfur-containing molecular formula. The experimental information provided defined that mass accuracy “*should be below 5 ppm*”, though did not guarantee this mass accuracy in the view of the authors. Therefore, the workflows were operated with a mass accuracy of 10 ppm and produced a second molecular formula (C_19_H_17_NO_4_), which was present in the trimMMD_sortAmass.txt file applied in workflow 2. This molecular formula was submitted. The correct molecular formula was submitted. The fragmentation mass spectrum acquired at 30eV was submitted to MetFrag, applying KEGG as the chosen database. MS/MS data at three collision energies were provided, data acquired at 30eV was chosen, as they provided the greatest number of product ions to most accurately define the structure of the metabolite. Two metabolites were reported, rutacridone epoxide and stylopine, with *in silico* fragmentation data matching to 20 experimentally-derived product ions for the former and 10 for the latter metabolite. Both of these metabolites were submitted to the challenge with scores of 1.0 and 0.5, respectively. The correct molecular structure was not submitted.

#### 2.2.5. Challenge 5

The four *m/z* values defined in the challenge as being detected in positive ion mode were analysed applying workflows 1 and 2 of the PUTMEDID_LCMS collection of workflows with a mass accuracy of 10 ppm (the experimental notes defined a mass accuracy of 5 ppm, though the results from challenge 4 showed a mass accuracy of 10 ppm was appropriate). The results showed that the [M+H]^+^ ion was detected and reported two molecular formula of C_19_H_23_NO_4_ and C_16_H_27_NO_4_S, which were present in the trimMMD_sortAmass.txt file applied in workflow 2. However, the relative isotopic abundance data showed no evidence of a sulfur-containing molecular formula; so, C_16_H_27_NO_4_S was removed, and C_19_H_23_NO_4_ was submitted to the contest. The correct molecular formula was submitted. This molecular formula and the fragmentation mass spectrum acquired at 10 eV were submitted to MetFrag, applying KEGG as the chosen database. MS/MS data were acquired at two collision energies; the data acquired at 20eV appeared to be inaccurate, as the highest *m/z* reported was greater than the molecular weight of the metabolite, and therefore, the data acquired at 10 eV data was applied. Five metabolites were reported; four metabolites matched two experimentally-derived product ions (of a possible 16) to *in silico*-derived product ions, and the one metabolite reported one product ion match. The latter metabolite was removed, because of the lower number of matches, and the four metabolites were submitted to the contest. As confidence in these four metabolites was not high, because only two of 16 product ions were matched, all were reported with the same score, as no discrimination in confidence could be obtained. The correct molecular structure was submitted.

#### 2.2.6. Challenge 6

The four *m/z* values defined in the challenge as being detected in positive ion mode were analysed applying workflows 1 and 2 of the PUTMEDID_LCMS collection of workflows with a mass accuracy of 10 ppm (the experimental notes defined a mass accuracy of 5 ppm, though the results from challenge 4 showed a mass accuracy of 10 ppm was appropriate). The results showed that the [M+H]^+^ ion was detected and reported two molecular formula of C_21_H_21_NO_6_ and C_14_H_25_NO_11_, which were present in the trimMMD_sortAmass.txt file applied in workflow 2. An error by our team was not to assess the carbon relative isotopic abundance, as had been performed in other challenges and which would have removed the C_14_H_25_NO_11_ option. Instead, C_21_H_21_NO_6_ and C_14_H_25_NO_11_ were submitted to the contest with scores of 0.5 and 1.0. The correct molecular formula was submitted as the second ranked possible molecular formula. These molecular formulae and the fragmentation mass spectrum acquired at 20 eV were submitted to MetFrag, applying KEGG as the chosen database. MS/MS data for three collision energies were available; data acquired at 20 eV data was chosen, as this included a *m/z* peak representing the molecular ion and which the authors prefer to observe in MS/MS data. Seven metabolites were reported; four metabolites were reported with a molecular formula of C_21_H_21_NO_6_, and three metabolites were reported with a molecular formula of C_14_H_25_NO_11_. The three latter metabolites matched a greater number of experimentally-derived product ions to *in silico*-derived product ions. These three metabolites were submitted to the contest with the MetFrag calculated scores. The correct molecular structure was not submitted.

#### 2.2.7. Challenge 10

The three *m/z* values defined in the challenge as being detected in positive ion mode were analysed applying workflows 1 and 2 of the PUTMEDID_LCMS collection of workflows with a mass accuracy of 5 ppm (as would be expected with a hybrid LTQ-Orbitrap mass spectrometer). The results showed that the [M+H]^+^ ion was detected and reported a single molecular formula of C_14_H_9_NO_2_, which was present in the trimMMD_sortAmass.txt file applied in workflow 2. The correct molecular formula was submitted. This molecular formula and the fragmentation mass spectrum (MS/MS data were only acquired at one collision energy of 10eV data) were submitted to MetFrag, applying KEGG as the chosen database. Three metabolites were reported; one metabolite matched *in silico* fragmentation data to two experimentally-derived product ions, whereas two metabolites matched one product ion. As the number of matches was low and none were conclusive, all three metabolites were submitted to the contest. The correct molecular structure was not submitted.

#### 2.2.8. Challenge 13

The three *m/z* values defined in the challenge as being detected in positive ion mode were analysed applying workflows 1 and 2 of the PUTMEDID_LCMS collection of workflows with a mass accuracy of 5 ppm (as would be expected with a hybrid Orbitrap mass spectrometer). The results showed that the [M+H]^+^ ion was detected and reported a single molecular formula of C_9_H_16_N_4_O_7_, which was present in the trimMMD_sortAmass.txt file applied in workflow 2. The correct molecular formula was not submitted. Further research after the results were released shows that the correct molecular formula (C_19_H_17_OP) is not present in the reference file applied in workflow 2. The molecular formula C_9_H_16_N_4_O_7_ and the fragmentation mass spectrum collected applying collision-induced dissociation (CID) at a normalized collision energy (NCE) of 45% were submitted to MetFrag, applying KEGG as the chosen database. Four MS/MS datasets were available, CID at 45 and 75% and higher-energy C-trap dissociation (HCD) at 45 and 75%. CID at 45% was chosen, as it provided as many product ions as the other data provided, though HCD at 45% provided the same number of product ions. No matches were reported, and the process was repeated applying ChemSpider. Three metabolites were reported; one metabolite matched *in silico* fragmentation data to five experimentally-derived product ions, whereas two metabolites matched two product ions. The former metabolite (N-hydroxy-6-(hydroxyamino)-5,6-dihydrocytidine) was submitted to the contest, as this showed a significantly better score in MetFrag. The correct molecular structure was not submitted, because the correct molecular formula was not applied.

#### 2.2.9. Challenge 14

The two *m/z* values defined in the challenge as being detected in positive ion mode were analysed applying workflows 1 and 2 of the PUTMEDID_LCMS collection of workflows with a mass accuracy of 5 ppm (as would be expected with a hybrid Orbitrap mass spectrometer). The results showed that the [M+H]^+^ ion was detected and reported a single molecular formula of C_12_H_9_N, which was present in the trimMMD_sortAmass.txt file applied in workflow 2. The correct molecular formula was submitted. To the team, this appeared to be related to a chemical rather than a metabolite, and therefore, ChemSpider, and not KEGG, was applied in MetFrag. This molecular formula and the fragmentation mass spectrum collected applying HCD at 180V were submitted to MetFrag applying ChemSpider as the chosen database. MS/MS data were provided at two different collision energies, and the data acquired at 120 V was chosen, as these MS/MS data provided the greatest number of product ions to allow structural information to be deduced most accurately. Sixty-five chemicals were reported; many of these were defined as chemically unusual, as they contained C-N triple covalent bonds or C-C triple covalent bonds or three fused benzene rings or two fused C-C double bonds. These chemicals were removed to leave 23 possible molecular structures. The 23 molecular structures were submitted to the contest with MetFrag scores. The correct molecular structure was submitted and was ranked as 12^th^ in possible molecular structures.

#### 2.2.10. Challenge 15

The two *m/z* values defined in the challenge as being detected in positive ion mode were analysed applying workflows 1 and 2 of the PUTMEDID_LCMS collection of workflows with a mass accuracy of 5 ppm (as would be expected with a hybrid Orbitrap mass spectrometer). The results showed that the [M+H]^+^ ion was detected and reported a single molecular formula of C_12_H_13_NO_2_, which was present in the trimMMD_sortAmass.txt file applied in workflow 2. The correct molecular formula was submitted. This molecular formula and the fragmentation mass spectrum collected, applying HCD at a 120V, were submitted to MetFrag, applying KEGG as the chosen database. MS/MS data were acquired at two different collision energies; both provided the same number of product ions, and the data acquired at 120V was chosen. Three metabolites were reported; one metabolite matched *in silico* fragmentation data to 10 experimentally derived product ions, whereas the other two metabolites matched one and no product ions. The former metabolite (indole-3-butyric acid) was submitted to the contest, as this showed a significantly higher score in MetFrag. The correct molecular structure was not submitted. The submitted and correct structure had the same sub-structure (indole), the additional substructures were different for the correct and submitted structures.

#### 2.2.11. Challenge 17

The three *m/z* values defined in the challenge as being detected in positive ion mode were analysed applying workflows 1 and 2 of the PUTMEDID_LCMS collection of workflows with a mass accuracy of 5 ppm (as would be expected with a hybrid Orbitrap mass spectrometer). The results showed that the [M+H]^+^ ion was detected and reported a single molecular formula of C_13_H_13_N_3_, which was present in the trimMMD_sortAmass.txt file applied in workflow 2. The correct molecular formula was submitted. This molecular formula and the fragmentation mass spectrum collected applying HCD 90V (CID and HCD data were provided; HCD data provided more product ions) were submitted to MetFrag, applying KEGG as the chosen database. MS/MS data were provided applying two different fragmentation techniques (CID and HCD); the data acquired applying HCD was chosen, as these MS/MS data provided the greatest number of product ions to allow structural information to be deduced most accurately. Three metabolites were reported; only one metabolite matched *in silico* fragmentation data to experimentally-derived product ions, whereas the other two metabolites matched no product ions. The former metabolite (3-amino-1,4-dimethyl-5H-pyrido[4,3-b]indole) was submitted to the contest. The correct molecular structure was not submitted. The correct structure was similar to the submitted structure, as both contained two sub-structures that were identical (benzene and aniline), though the difference between both structures was the chemical sub-structure connecting these two sub-structures. 

### 2.3. Discussion

Applying two separate workflows to putatively annotate metabolites was an enjoyable process and tested the authors’ knowledge of chemistry, metabolites and metabolite annotation applying automated workflows and manual interpretation. The results presented here were acquired, applying one workflow for each challenge. However, different workflows were also assessed, but were not submitted to the CASMI contest, because of the opportunity to submit only one result for each challenge. Other workflows investigated included MI-Pack [14] and Mass Frontier [27], both showed good results, but will not be discussed further here. The authors were ranked as first in the contest for category 1; they submitted results to 11 challenges, of which their highest probability match was correct in eight challenges, their second highest probability match was correct in one challenge and their submission was not correct in two challenges. Of these two challenges providing incorrect submissions, the mass error of the metabolite was higher than reported in the contest information for challenge two (>30 ppm compared to an expected mass accuracy of 5 ppm). This highlights an important point that the mass accuracy of any mass spectrometer does not always meet the specifications provided by instrument companies, caused by either analyst error (including mass calibration errors) or inadequate ion populations to provide accurate determination of the ion peak shape and apex. For challenge 13, the molecular formula of the correct metabolite was not present in the trimMMD_sortAmass.txt file applied in workflow 2 of PUTMEDID_LCMS. The authors did not submit entries for three challenges (11, 12 and 16), as in-source fragmentation was present, and it is known that PUTMEDID_LCMS does not report accurate molecular formula for metabolites undergoing uncommon in-source fragmentation (though it operates well for loss of H_2_O, HCO_2_H and NH_3_). The results submitted to category 1 have shown that the PUTMEDID_LCMS operates very well in defining the molecular formula; in only two of thirteen submissions were the results not correct, one due to a limitation of the reported data and one due to a limitation of a reference file applied in PUTMEDID_LCMS.

The authors’ accuracy in defining chemical structures in category 2 was significantly lower than for category 1; they submitted results to 11 challenges, of which their highest probability match was correct in one challenge (challenge 1), their submission was not correct in eight challenges and, in two challenges, the correct structure was ranked by the authors as fourth (challenge 5) and 12th (challenge 14). All challenges were performed, with the belief that all chemicals were endogenous or exogenous metabolites, and this logic was applied in the processes employed to define molecular structure. In the eight challenges where the submission was not correct, the authors applied only a metabolite-specific database (KEGG) or in challenges where there were no matches to KEGG, ChemSpider was applied, but results were filtered to remove chemicals (by a single author, W.B.D.) not believed to be derived from endogenous and exogenous metabolism. This logic is applied in all metabolomics studies by the authors, though the contest does not state anywhere that chemicals are endogenous or exogenous metabolites, and so, applying this logic was not appropriate. Here, the application of chemical rather than metabolite-specific libraries when integrated with the application of MetFrag would be expected to provide greater accuracy in the annotation of metabolites, though this has not been experimentally assessed by the authors.

This observation highlights an important aspect of the annotation process. The search space for chemicals is very large; PubChem contains more than 31 million entries [19]. The metabolite search is a sub-component of the chemical search space and is smaller than the chemical search space, though, depending on the biological sample, it can be comprised of thousands of unique metabolite structures. For example, yeast, plant and human metabolic reconstructions contain only hundreds or thousands of metabolites [29,30,31], whereas some other databases contain over 40,000 metabolites (e.g., HMDB [21]). Some metabolites are not specific to a single organism or biological sample, whereas other metabolites can be specific to a single organism or biological sample. Some databases are specific to chemicals and are large (for example, PubChem [19]), whereas some databases are metabolite-specific (for example, KEGG [20,32]). To provide accuracy in the annotation process, applying information on the organism and environment will always be beneficial. For example, when performing a metabolite search in human biofluids, you would include drugs and their metabolites in the search space, whereas in plants and microbes, you would not, unless they were specifically added to the environment. Following on from the previous discussion, the choice of database or databases to apply is important. Again, this should be organism-specific if the organism or biological sample is known, so as to reduce the search space and number of returned hits, and a greater number of organism-specific databases are being constructed. However, when information is limited and the complexity of samples is high, then chemical rather than metabolite databases should be applied, reducing the specificity of the search by increasing the number of possible matches. This was the case for the CASMI challenge, as no information was provided on the origin of the biological sample, or for challenges based on single authentic chemical standards; no information was provided on the biological sample type where the chemical is expected to be observed. This logic can also be applied for mass spectral library searches, specifically the decision of whether to apply metabolite-specific (for example, METLIN [25] HMDB [21] and MassBank [26]) or chemical-specific mass spectral libraries (for example, the NIST12 MS/MS database [33]).

The authors choose to apply *in silico* fragmentation to aid in their putative annotation process, as they are interested in this process for the annotation of metabolites not present in mass spectral libraries and the appropriateness of applying this process. MetFrag was chosen to perform *in silico* fragmentation, as it was freely available to the academic research community. The authors also applied Mass Frontier, a commercial software package available from HighChem, though did not submit any results from these data, as only single submissions were available for each challenge. A second process, which the authors apply, is to submit experimentally-derived MS/MS data to freely available mass spectral MS/MS libraries, including METLIN [25], MassBank [26] and HMDB [21]. However, because of the interests of the authors, we decided to submit the *in silico*-derived data from MetFrag only for this contest.

The authors would like to emphasise that all data presented here for unknowns (but not challenges based on chemical standards) are provided as putative annotations (level 2, according to the MSI [34]). To provide level 1 identifications, authentic chemical standards would need to be purchased and data acquired applying the same analytical methods.

## 3. Experimental Section

### 3.1. Derivation of Molecular Formulae from Experimentally Determined m/z

PUTMEDID_LCMS [13] was applied as the first process to derive single or multiple molecular formulae, which exhibited a match to the experimentally determined *m/z* and associated mass accuracy (applied as +/− mass accuracy, ppm). The data provided in the challenge included data on experimentally determined *m/z* values, retention times and responses. These data were inserted in to a Microsoft Excel worksheet as follows; *m/z* values for each metabolite feature were inserted in to column 1, the experimentally determined retention time was inserted in to column 2 (or a value of 100 was inserted for direct infusion studies) for each metabolite feature and the response data was inserted in to columns three to twelve. The insertion of multiple columns related to response allowed correlation analysis to be performed in workflow 1, below.

Workflow 1 and workflow 2 of the three available workflows in the PUTMEDID_LCMS package were applied, as defined in the standard operation procedure (SOP), available at [35]. Workflow 1 provided an output file, which defines pairwise correlation coefficients between different metabolite features. The output file was imported in to workflow 2, which applies correlation coefficient results, ion mode, *m/z* differences calculated between metabolite features, retention time and median peak area to group together and annotate metabolite features (adducts, dimers, isotopes) derived from the same metabolite. From these data, the molecular weight of the non-charged metabolite was calculated and matched to the molecular weight of single or multiple molecular formulae present in a reference file (trimMMD_sortAmass.txt). The mass accuracy defined in the contest or a mass accuracy defined by the author (W.B.D.) was applied in workflow 2 to limit the search space. Where multiple molecular formulae were present, the different options were normally (but not always) assessed, applying relative isotope abundance calculations for carbon and/or sulfur, and inaccurate molecular formulae were removed. Where molecular formula contained sulfur atoms, relative isotopic abundances were assessed to determine whether these data indicated the presence of sulfur atoms and the number of sulfur atoms present; if their presence was not supported by these isotopic data, then further work was performed, as defined for each challenge.

In challenges where the process above applying PUTMEDID_LCMS provided no molecular formula matches, one of two processes was performed, as chosen by the authors. The first process assessed the experimental information provided and reduced the mass accuracy applied in workflow 2 of the PUTMEDID_LCMS process. The second process applied MetFrag (v 0.9, [15,36]) to perform a molecular formula search applying the neutral mass of the metabolite, as calculated by PUTMEDID_LCMS, KEGG as the chosen database, and a mass accuracy defined in the results section.

The molecular formula or formulae chosen by the authors were manually copied to a .txt file, and a score was applied, ranging from 0.0 to 1.0, with values of 1.0 describing the highest probability of reporting an accurate putative annotation.

### 3.2. Derivation of Chemical Structure from Gas-Phase Fragmentation Mass Spectra and MetFrag

MetFrag (v0.9, [15,36]) was applied to derive putative molecular structures, specifically by searching two databases (KEGG [20] and ChemSpider [18]), applying the single or multiple molecular formulae to identify putative molecular structures, followed by *in silico* fragmentation of these molecular structures and matching of these *in silico* data to experimentally-derived gas-phase fragmentation mass spectra provided in the contest. KEGG was applied as the only database in cases where matches to the inputted molecular formula were observed and biologically-related metabolites were suspected to be present. ChemSpider was applied in challenges where no matches were observed to the KEGG database or where chemicals, which were not biologically-related, were suspected to be present. Search ppm was set at 5 ppm, unless stated otherwise; the “Only biological compound” option was applied; the “Limit # of structures” was set at 2,000; and m/z ppm was set at 10 ppm, unless stated otherwise. Experimentally-derived MS/MS data was manually copied into MetFrag; where multiple sets of MS/MS data were available, one was chosen as optimal, based on providing a range of product ions, but not greater than 30 product ions. The reported metabolites and the number of product ions matched were manually assessed to determine whether all were potential molecular structures matching to the data; structures were removed, which the authors defined as not being biologically-related or where structures showed lower numbers of matches to fragment ions compared to other molecular structures.

The InChI for each molecular structure was manually acquired from one of two databases (ChemSpider [18] and PubChem [19]) and were manually copied to a .txt file. A confidence score was applied (taken from the MetFrag result or defined manually), ranging from 0.0 to 1.0, with values of 1.0 describing the highest probability of reporting an accurate molecular structure.

## 4. Conclusions

Researchers at The University of Birmingham took part in the CASMI open contest and submitted responses to 11 challenges, each in category 1 and 2. When applying the PUTMEDID_LCMS workflows to category 1 challenges, a high level of accuracy was observed, with nine of eleven submissions correct; the two incorrect submissions being related to a significantly lower mass accuracy being observed experimentally than was expected and reported and being related to the absence of the correct molecular formula in the molecular formula reference file applied in the workflows. When employing processes applied by the authors in metabolomic studies to eleven category 2 challenges, a low level of accuracy was reported, with eight challenges not submitting the correct structure and three challenges submitting the correct structure ranked by the authors as first, fourth and twelfth. The results for category 2 highlight the importance of knowing the organism or biological sample from which data has been acquired; this aids in focusing the chemical or metabolite search space applied; the authors here assumed all metabolites were of a biological origin, which was not the case.
